# Molecular dynamics simulation study of doxorubicin adsorption on functionalized carbon nanotubes with folic acid and tryptophan

**DOI:** 10.1038/s41598-021-03619-8

**Published:** 2021-12-20

**Authors:** Tahereh Arabian, Sepideh Amjad-Iranagh, Rouein Halladj

**Affiliations:** 1 Department of Chemical Engineering, Amirkabir University of Technology, Tehran, Iran; 2 Department of Materials and Metallurgical Engineering, Amirkabir University of Technology, Tehran, Iran

**Keywords:** Biomaterials - proteins, Computational methods, Atomistic models, Drug delivery, Carbon nanotubes and fullerenes

## Abstract

In this work, molecular dynamics (MD) simulation is used to study the adsorption of the anticancer drug, doxorubicin (DOX), on the wall or surface of pristine and functionalized carbon nanotubes (FCNTs) in an aqueous solution. Initially, the CNTs were functionalized by tryptophan (Trp) and folic acid (FA), and then the DOX molecules were added to the system. The simulation results showed that the drug molecules can intensely interact with the FCNTs at physiological pH. Furthermore, it was found that as a result of functionalization, the solubility of FCNTs in an aqueous solution increases significantly. The effect of pH variation on drug release from both pristine and FCNTs was also investigated. The obtained results indicated that in acidic environments due to protonation of functional groups (Trp) and as a result of repulsive interaction between the DOX molecule and functional groups, the release of DOX molecules from FCNT’s surface is facilitated. The drug release is also strongly dependent on the pH and protonated state of DOX and FCNT.

## Introduction

Nanomaterials have dimensions on the nanometer scale and are comparable with biological molecules that are constituents of living systems^1^. They can be used in biomedical applications such as drug delivery, chemotherapy, radiation therapy, and biosensors^2,3^. Carbon nanotubes have been recognized as a good choice for biological and biomedical applications due to their excellent electrical and mechanical properties and have been used extensively in the last decade^4–7^. Single-walled carbon nanotubes (SWCNTs) are hollow cylinders about 0.5 to 5 nm in diameter made of rolled graphite sheets^8–11^. Pristine carbon nanotubes are toxic and hydrophobic, limiting their biomedical and biotechnological applications. Therefore, to overcome these limitations, the surface of carbon nanotubes can be modified. In general, surface modification is done both covalently and non-covalently and using biological molecules^12–14^. Bio functionalization of the CNT’s surface via amino acids can provide them with proton donor–acceptor characteristics since amino acids contain both proton donor carboxylic acid (COOH) and the proton acceptor amine (NH2) groups^15–18^. Various studies have been performed on the CNTs functionalized with amino acids. Mallekpour et al.^19^ studied the covalent surface functionalization of multi-walled carbon nanotubes (MWCNTs) with different natural amino acids, and their results indicated that the formation of amino acid on the MWCNTs with availability for further chemical operation whereas the structure of MWCNTs remained relatively intact. Rahmani et al.^20^ studied the adsorption of two amino acids on the CNTs surface to investigate their effect on the solvation properties of CNTs. The complex formation of alanine and histidine with the armchair single-wall carbon nanotube (SWCNT) was studied by density functional theory (DFT). The results of computer simulation in an aqueous solution indicate that amino acid functionalization increases the intermolecular interactions between carbon nanotube and water molecules. On the other hand, a method to increase the adsorption and targeting capacity of CNT is modifying their surface with specific ligands^21^.

One of the ligands used for this purpose is folic acid (FA). Folate receptors are present on the surface of many cancer cells, so using this ligand is a way to target cancer cells^22–26^. It is an essential vitamin from the B vitamin group (B9)^27,28^. Recently, studies have been performed on the interactions of carbon nanotubes and folic acid. Tavakolifard et al.^29^ functionalized SWCNT covalently with paclitaxel (PTX), an anticancer drug, and folic acid (FA) as a targeting agent for many tumors. The results showed good conjugation of the targeting molecule and the anticancer drug on the surface of the CNT. Mehra et al.^30^ assessed and compared the in vitro and in vivo cancer targeting propensity of DOX loaded FA, and estrone (ES) anchored PEGylated multi-walled carbon nanotubes (MWCNTs). Ellison et al.^31^ functionalized SWCNT covalently with FA Infrared spectroscopy confirmed intact molecular binding to the SWNTs through the formation of an amide bond between a carboxylic acid group on SWCNT and the primary amine group of FA. Carbon nanotubes are introduced as promising carriers for the treatment of cancer. Because they increase the circulation time of the drug, reduce systemic toxicity, and increase the accumulation of the drug at the tumor site^32^. Anticancer drugs can be combined with carbon nanotubes through covalent or non-covalent interactions^33,34^. DOX is a chemotherapy drug of the anthracycline class of anticancer drugs^35,36^. The drug release at the target site is sometimes additionally triggered by using various physical factors like infrared radiation, and ultrasonic and magnetic fields. Biochemical triggering factors of which pH change is appropriate from neutral to acidic state occurring in tumor tissue have also been considered^37,38^.

In the present molecular dynamics studies^39–42^, the adsorption and release of DOX have been investigated for CNT, functionalized with FA and tryptophan (Trp) (a non-polar aromatic amino acid)^43^ in both neutral and acidic pH.

## Materials and methods

### Structure preparation

**Figure 1:**
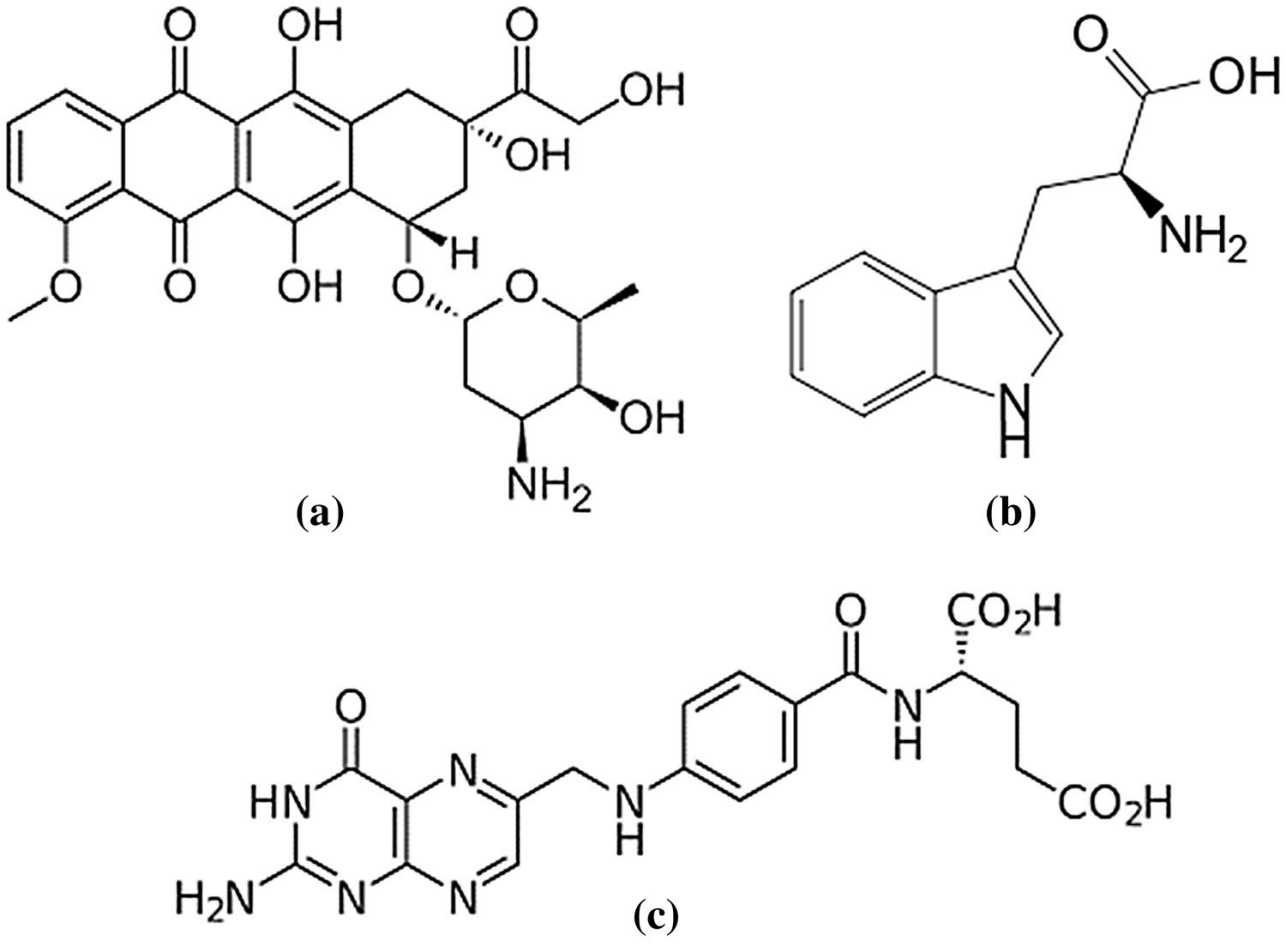
The initial structure of (**a**) Doxorubicin (**b**) Tryptophan (**c**) Folic acid.

In the first step, an armchair (12, 12) SWCNT with a diameter of 16.283 Å and length of 40 Å was constructed as the model for DOX-CNT drug carrier system. The CNT model was generated by employing the Nanotube Modeler package^44^. The initial structure of DOX was obtained from the DRUGBANK server^45^. The partial charge of all atoms in DOX molecules was obtained by applying the electrostatic potential (ESP) method^46^ by the DFT, 6-31G (d,p), B3LYP utilizing GAMESS^47^ software. The chemical structure of Trp and FA were extracted from the PubChem website^48^. The molecular structure of DOX, Trp, and FA are shown in Fig. 1.

**Figure 2:**
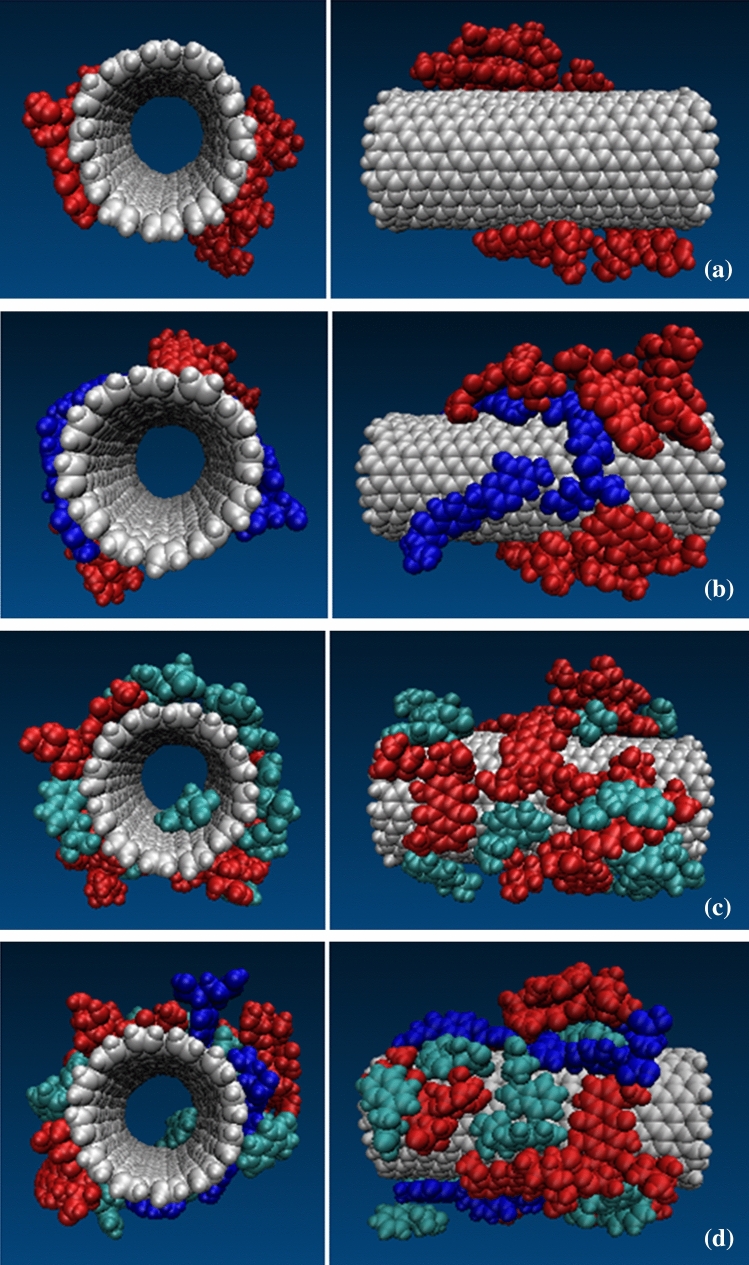
The structures of the functionalized CNTs. (**a**) DOX (red)-CNT; (**b**) DOX (red)-Trp (green)-CNT; (**c**) DOX (red)-FA (blue)-CNT; (**d**) DOX (red)-Trp (green)-FA (blue)-CNT.

CNTs were functionalized with both Trp and FA. Also Table 1 presents systems and structures used in the simulation boxes. The structures of the functionalized CNTs are shown in Fig. 2.

**Table 1:** Systems used in the simulation boxes.

Systems	CNT	Functional group	Drug
Drug-CNT	pristine CNT	0	8
Drug-Trp-CNT	CNT functionalized with Trp	20 Trp	8
Drug-FA-CNT	CNT functionalized with FA	5 FA	8
Drug-Trp-FA-CNT	CNT functionalized with Trp and FA	20 Trp and 5 FA	8

### Molecular dynamic simulation

The GROMACS package (version 2019.3)^49–51^ with the force field CHARMM27^52,53^ was used for the MD simulations. The parameters of the force field for the drug, Trp and FA molecules were obtained from the SwissParam website^54^. The dimensions of the simulation boxes were 5 × 5 × 5 nm. The water molecules were modeled by using the tip3p model^55^, and 3295 water molecules to the DOX-Trp-FA-CNT system, 3362 water molecules to the DOX-Trp-CNT system, 3458 water molecules to the DOX-FA-CNT system, and 3351 water molecules to the DOX-CNT system were added. During these MD simulations, the temperature was maintained at 300 K by employing a V-rescale thermostat^56^, and the pressure was held at 1 bar by applying the Parrinello–Rahman algorithm^57,58^. The Leap–Frog integration algorithm was utilized to solve the equation of motion under periodic boundary conditions in all directions. The cut-off distance for the van der Waals interactions was 1.3 nm. The electrostatic interactions were computed with the particle mesh Ewald method^59,60^. The Trp and FA molecules were randomly placed in the simulation boxes. First, tryptophan molecules were added to the box, and after complete absorption, folic acid molecules were added to absorb on the carbon nanotube's surface. Simulations of the absorption of these functional groups were performed for 10 ns. Then, 8 drug molecules (DOX) were added to each simulation boxes and the final simulations for each system were run for 40 ns to fully absorb the drug molecules. The visual molecular dynamics (VMD 1.9.4)^61^ software was used to visualize the studied systems.

### pH effect

In physiological application, two values of pH are important: pH = 7.4 and pH = 4–5.5, which are for normal conditions and cancerous cell environments, respectively. It was observed that the amount of the protonated state of Trp molecules as a functional group of CNTs DOX molecules increases in the acidic condition. To model the pH-controlled drug loading and then its release from the carrier on the cancerous cell, the number of Trp molecules in the protonated form (that is, changing of NH2 to NH3+) is of significant importance. DOX has an NH_2_ group with pKa = 8.6, and tryptophan has an NH_2_ group with pKa = 2.46.

In this study, the number of protonated Trp molecules was changed from 0 to 10 and 20. The MD simulation for each system was run for a period of 10 ns.

## Results and discussion

### Equilibration

At the first step, the equilibrium state and the stability of the simulation boxes were evaluated by observing the change in total energy and root mean square displacements (RMSD) of the simulation systems. Supplementary Figs. S1, S2 (presented in the supporting information) show that the total energy curves and RMSD values for the investigated systems remained constant and reached the equilibrium state after 5 ns.

### Solubility

The purpose of the functionalization of CNTs is to improve the solubility of CNTs in aqueous solutions^62^. To evaluate the effect of functional groups on the solubility of carbon nanotubes, different parameters such as the number of hydrogen bonds (NHBS) these formed between functionalized CNTs and water molecules, the value of solvent accessible surface area (SASA), and the solvation free energies calculated^12^. The solvation free energy is calculated using the following equation:

1 $$\Delta {G}_{s}=\sum_{atoms i}(\Delta \sigma \left(i\right)\sum ({A}_{i}-{A}_{i}^{r})),$$

where $\Delta \sigma \left(i\right)$ is the atomic solvation parameter, ${A}_{i}$ is the solvent-accessible surface area of an atom in the structure and ${A}_{i}^{r}$ is the solvent-accessible surface area of an atom in the reference state. A reference state is a standard state of a species in a phase limited to one particular pressure^63^.

The results reported in Table 2 indicate that the functionalized CNTs have higher SASA and higher number of hydrogen bonds but lower free energy than pristine CNT. The results show that the presence of functional groups increases solvent accessible surface areas for doxorubicin molecules. (as shown in Supplementary Figs. S3, S4 in the supporting information). Also, while no hydrogen bond formed between pristine CNT and water molecules, the number of hydrogen bonds between water molecules and the polar groups (–NH2, –COOH, –OH, and –O–) of the functionalized CNT increases up to almost 140 in the system (DOX-FA-Trp-CNT). It indicates the effect of a functional group on the interaction of functionalized CNT (FCNT) with water molecules as the solvent. As seen in Table 2, the increase in the SASA parameter from DOX-pristine CNT to DOX-FA-Trp-CNT occurs with a variation of NHB, indicating a lot more accessible surface for interaction FCNT is available.

**Table 2:** Computed solvent accessible surface area (SASA), number of hydrogen bonds and solvation free energy (SFE).

Systems	SASA (nm^2^)	NHB	SFE (kJ/mol)
DOX-CNT	51.13	0	298.68
DOX-Trp-CNT	62.52	65.55	224.79
DOX-FA-CNT	73.35	89.14	199.08
DOX-Trp-FA-CNT	81.47	140.49	160.07

### Interaction energies between the DOX and CNT/FCNTs

Leonard Jones and electrostatic interactions are two parts of interaction energies. The Lenard-Jones interaction is calculated from the following equation:

2 $${V}_{LJ}\left(r\right)=4\varepsilon \left[{\left(\frac{\sigma }{r}\right)}^{12}-{\left(\frac{\sigma }{r}\right)}^{6}\right],$$

where $r$ is the distance between two interacting particles, $\varepsilon$ is the depth of the potential well, and $\sigma$ is the distance at which the particle–particle potential energy $V$ is zero. The electrostatic interaction is calculated from the Coulomb’s equation:

3 $$\left|F\right|=K \frac{|{q}_{1}{q}_{2}|}{{r}^{2}},$$

where $K$ is Coulomb's constant ($K$ ≈ 8.988 × 109 N m^2^ C^−2^), ${q}_{1}$ and ${q}_{2}$ are two point charges, and $r$ is the distance between the charges.

**Figure 3:**
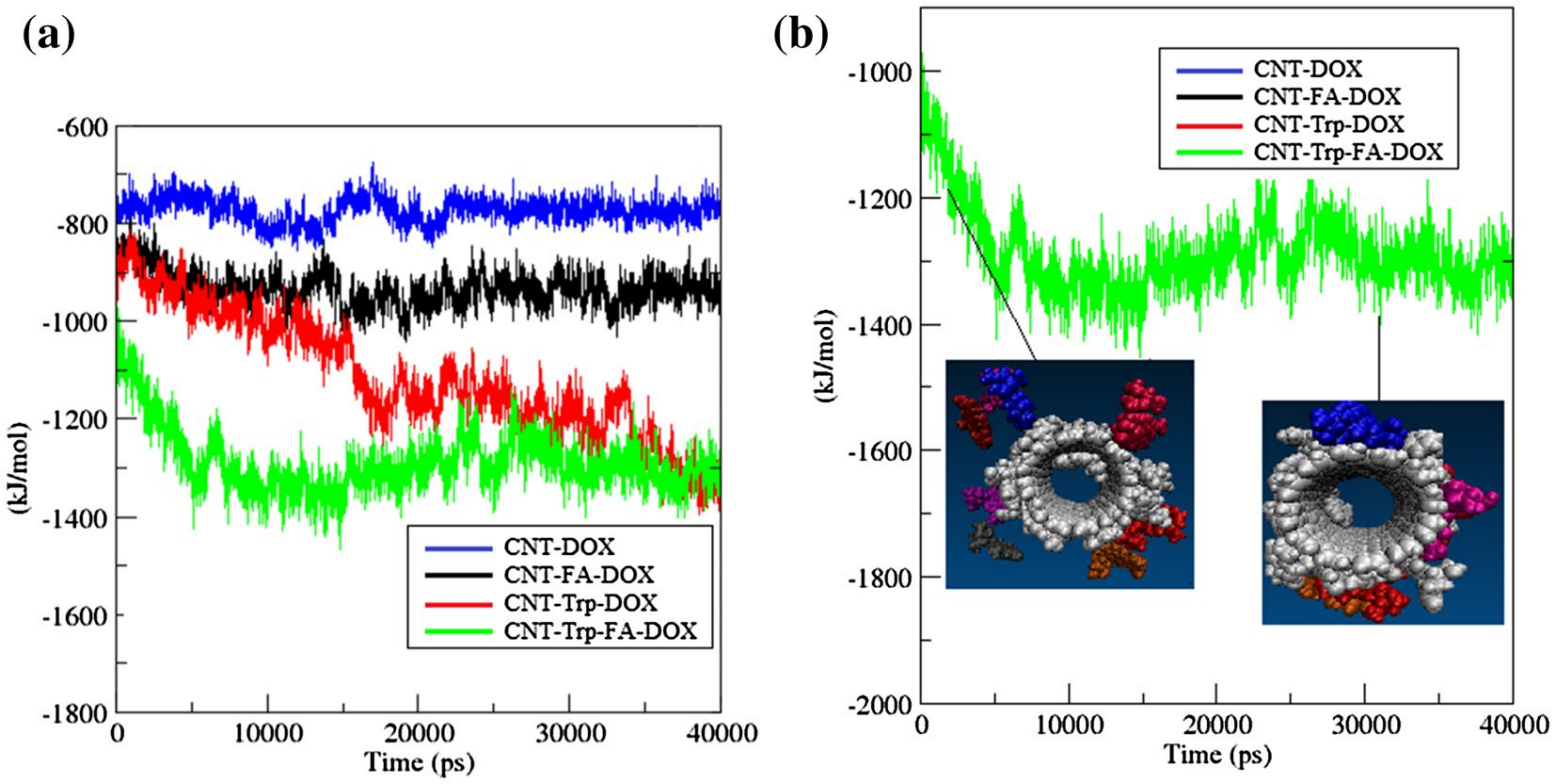
(**a**) van der Waals (Lennard–Jones) interactions between DOX molecules and CNT, FA-CNT, Trp-CNT and Trp-FA-CNT. (**b**) Snapshots of DOX adsorbed on Trp-FA-CNT surface.

The interaction energies between DOX molecules and the CNT/FCNTs were calculated to investigate the mechanism of DOX adsorption on the nanotube walls. Figure 3 shows the variation of van der Waals (vdW) interaction energies between DOX molecules and CNT/FCNTs. As seen in Fig. 3a, the vdW energies decrease as the simulation proceeds, which indicates that DOX molecules are adsorbed on the surface of the CNT/FCNTs. Figure 3b shows the process of adsorption of DOX molecules on the surface of Trp-FA-CNT. Also, as shown in Supplementary Fig. S5, the electrostatic interaction during the simulation is reduced by drug adsorption. Drug molecules and functionalized nanotubes interact through van der Waals and electrostatic interactions. At the beginning of the adsorption process, the electrostatic interaction is high but decreases during the simulation (see Supplementary Fig. S5 in the Supplementary Information). On the other hand, reducing the electrostatic interaction and overcoming the $\pi -\pi$ interaction between the aromatic rings of DOX and fCNT/CNT causes the drug molecules to be adsorbed on the fNT/CNT surface by vdW interaction. The calculated average vdW and electrostatic energies (kJ/mol) are reported in Table 3. In addition, the results show that the functionalization of CNTs has a significant effect on reducing vdW and electrostatic energies. Also, among the studied systems, the CNT functionalized with Trp and FA has the highest adsorption of the drug. Hasanzade et al.^64^ reported that the vdW interactions can be considered as the main interactions in the delivery of the DOX molecules. Tables 4 and 5 show that the average vdW and electrostatic energies between water and drug and FCNTs, respectively. These average values were obtained from the number of frames. Simulations were performed at 4000 frames. According to the tables, in FA-CNT, the drug molecules have stronger interactions with water. As a result, FCNTs represent higher solubility in water. In addition, the interaction of water molecules with the pristine CNT is higher than with the other systems.

**Table 3:** Average vdW and electrostatic energies (kJ/mol).

Systems	vdW (kJ/mol)	Electrostatic energy (kJ/mol)	Total energy (kJ/mol)
DOX-CNT	− 771.59	0	− 771.59
DOX-FA-CNT	− 933.83	− 156.834	− 1090.664
DOX-Trp-CNT	− 1113.41	− 226.634	− 1340.044
DOX-Trp-FA-CNT	− 1288.44	− 447.948	− 1736.388

**Table 4:** Average vdW and electrostatic energies (kJ/mol) between the DOX and water.

Systems	vdW (kJ/mol)	Electrostatic energy (kJ/mol)	Total energy (kJ/mol)
DOX-CNT	− 653.42	− 2222.26	− 2875.68
DOX-FA-CNT	− 710.20	− 2508.05	− 3218.25
DOX-Trp-CNT	− 619.26	− 2268.81	− 2888.07
DOX-Trp-FA-CNT	− 628.10	− 2290.75	− 2918.85

**Table 5:** Average vdW and electrostatic energies (kJ/mol) between the carriers and water.

Systems	vdW (kJ/mol)	Electrostatic energy (kJ/mol)	Total energy (kJ/mol)
DOX-CNT	− 2323.28	0	− 2323.28
DOX-FA-CNT	− 2241.38	− 1712.75	− 3954.13
DOX-Trp-CNT	− 2250	− 2509.12	− 4759.12
DOX-Trp-FA-CNT	− 2218	− 3852.57	− 6070.57

### Number of contacts and contact area

**Figure 4:**
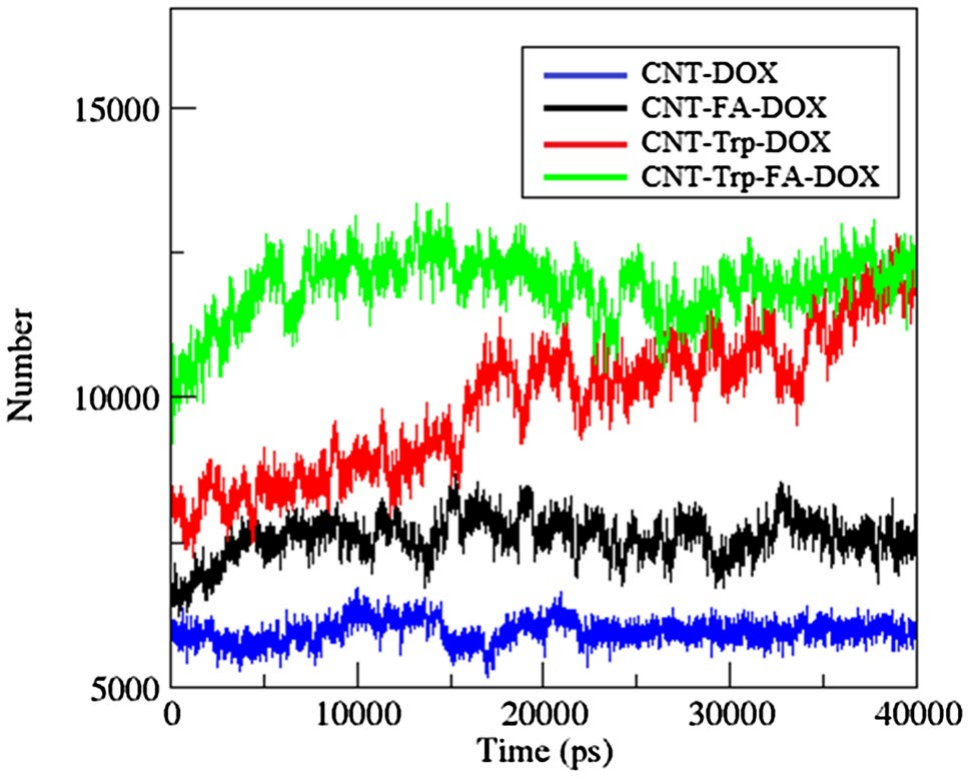
Number of atomic contacts between DOX and CNT, FA-CNT, Trp-CNT and Trp-FA-CNT.

The number of contacts that are formed between DOX and CNT/FCNT can be computed via the following expression:

4 $${N}_{C}\left(t\right)= \sum_{i=1}^{{N}_{CNT/FCNT}}\sum_{j=1}^{{N}_{DOX}}\underset{{r}_{i}}{\overset{{r}_{i}+0.6nm}{\int }}\delta \left(r\left(t\right)- {r}_{j}\left(t\right)\right)dr,$$

where ${N}_{CNT/FCNT}$ is the total numbers of atoms in the CNT/FCNT and ${N}_{DOX}$ is the total number of atoms in the drug, and ${r}_{i}$ is the distance of the *j*$\mathrm{th}$ atom of DOX from the *i*th atom of the CNT/FCNT. Figure 4 shows the number of atomic contacts between the DOX molecules and the pristine CNT and FCNTs. As this figure shows, the number of atomic contacts increases with the adsorption of drug molecules on the CNT/FCNT during the simulation. Then, at the end of the simulation, the number of the contacts of DOX-CNT, DOX-FA-CNT, DOX-Trp-CNT, and DOX-Trp-FA-CNT are 5971.89, 7581.11, 9893.74, and 11,895.41, respectively. These values indicate that the number of contacts between the DOX molecules and the carrier in the FCNT is higher than the pristine CNT, which shows the significant role of functional groups in drug adsorption on the carrier surface.

### Radial distribution function (RDF)

**Figure 5:**
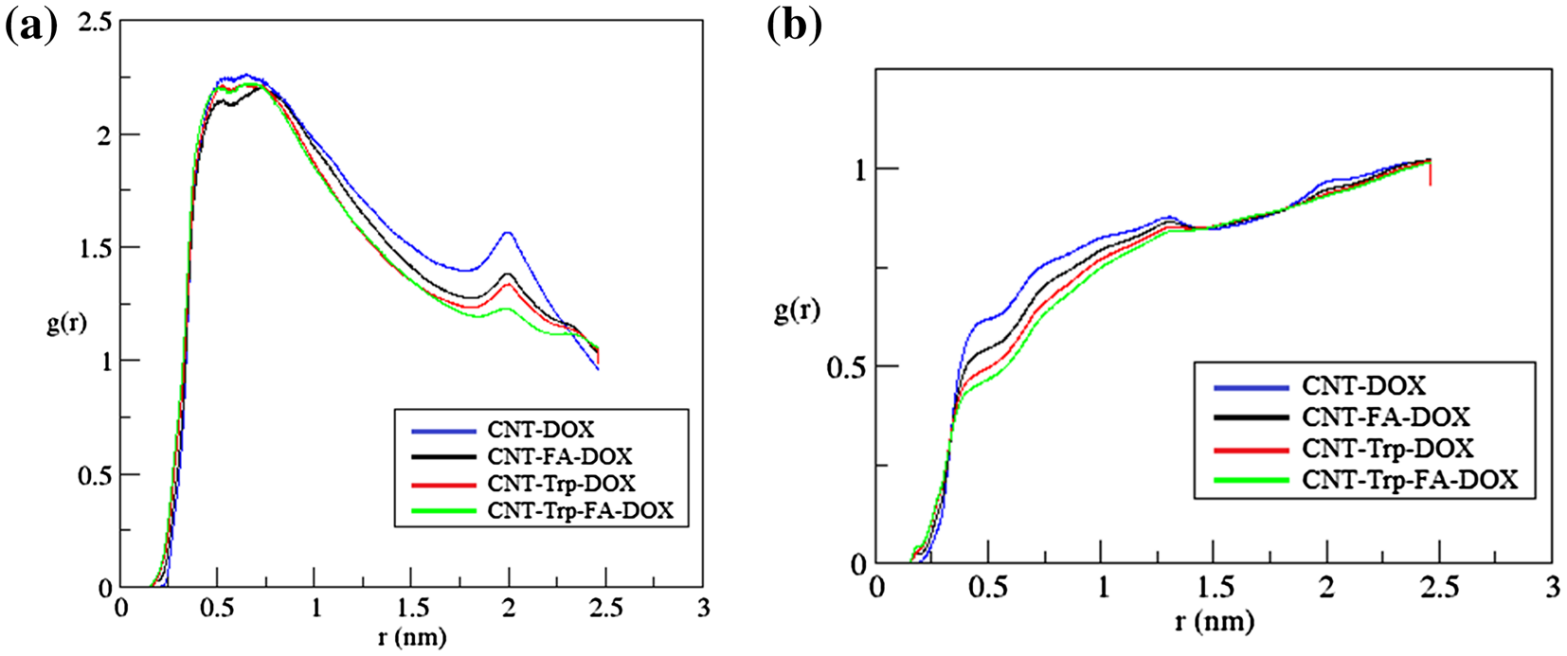
(**a**) Radial distribution functions (RDFs) plots for the drug molecules around CNT/FCNTs. (**b**) RDF plots for water molecules around the functional groups in FCNT systems.

**Figure 6:**
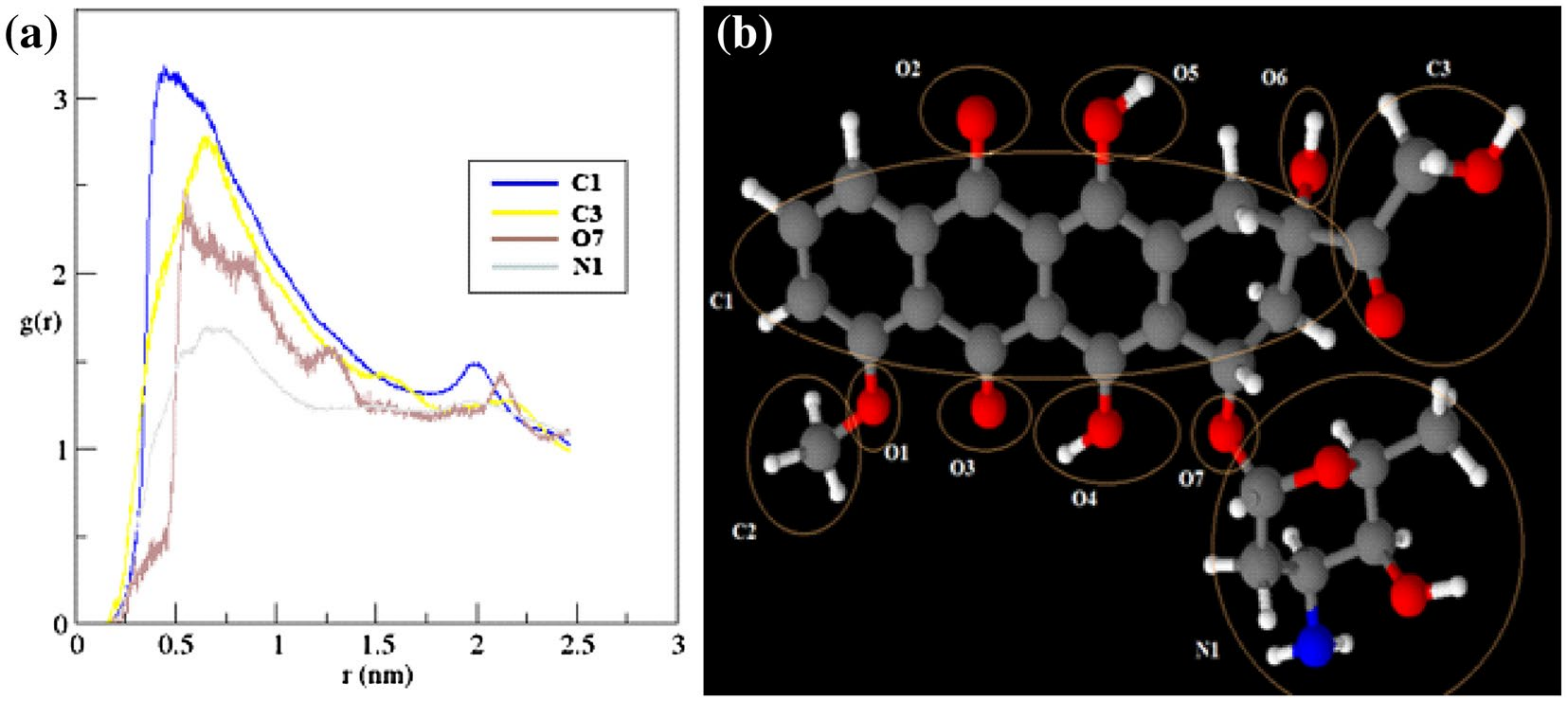
(**a**) RDFs between DOX atoms and Trp-FA-CNT. (**b**) Schematic model of atomic groups of a DOX molecule.

To examine the interactions between DOX and CNT/FCNTs, the radial distribution function (RDF) for all studied systems was calculated by using the following equation:

5 $${g}_{AB}\left(r\right)=\frac{\langle {\rho }_{B}(r)\rangle }{{\langle {\rho }_{B}\rangle }_{local}} \frac{1}{{\langle {\rho }_{B}\rangle }_{local}} \frac{1}{{N}_{A}} {\sum }_{i\in A}^{{N}_{A}}{\sum }_{j\in B}^{{N}_{B}}\frac{\delta ({r}_{ij}-r)}{4\pi {r}^{2}},$$

where $\langle {\rho }_{B}(r)\rangle$ is the partial density of component *B* at distance *r* from component *A* and ${\langle {\rho }_{B}\rangle }_{local}$ is the partial density of the average component *B* in all spheres around particles *A* with radius *r*^65^. Figure 5a shows that RDF curves have two peaks in the approximate range of 0.5 to 2.1 nm, indicating that these interactions between DOX and CNT/FCNTs have occurred in this range. The results also show that the strongest peak belongs to the Trp-FA-CNT. In addition, the RDF curve for water molecules located around each functional group was calculated. As Fig. 5b shows, more water molecules are located around Trp-FA-CNT than around FA-CNT, Trp-CNT, or pristine CNT. Therefore, it can be concluded that not only do the existence of a functional group of the DOX affect adsorption, but the type of it also influences the rate of adsorption. To understand the molecular orientation of DOX adsorbed on CNTs, we calculated the atomic RDF for DOX molecules, and the results are given in Fig. 6. As seen in Fig. 6a, the strongest peak belongs to the aromatic group of the DOX molecule. On the other hand, previous studies^66,67^ showed that these peaks are due to the formation of $\pi -\pi$ interactions between the aromatic ring of DOX molecules and the sidewall of CNTs or between drug molecules and the aromatic ring of functional groups. Figure 6b shows groups of atoms of a DOX molecule that interact with the surface of CNT.

### Drug release

**Figure 7:**
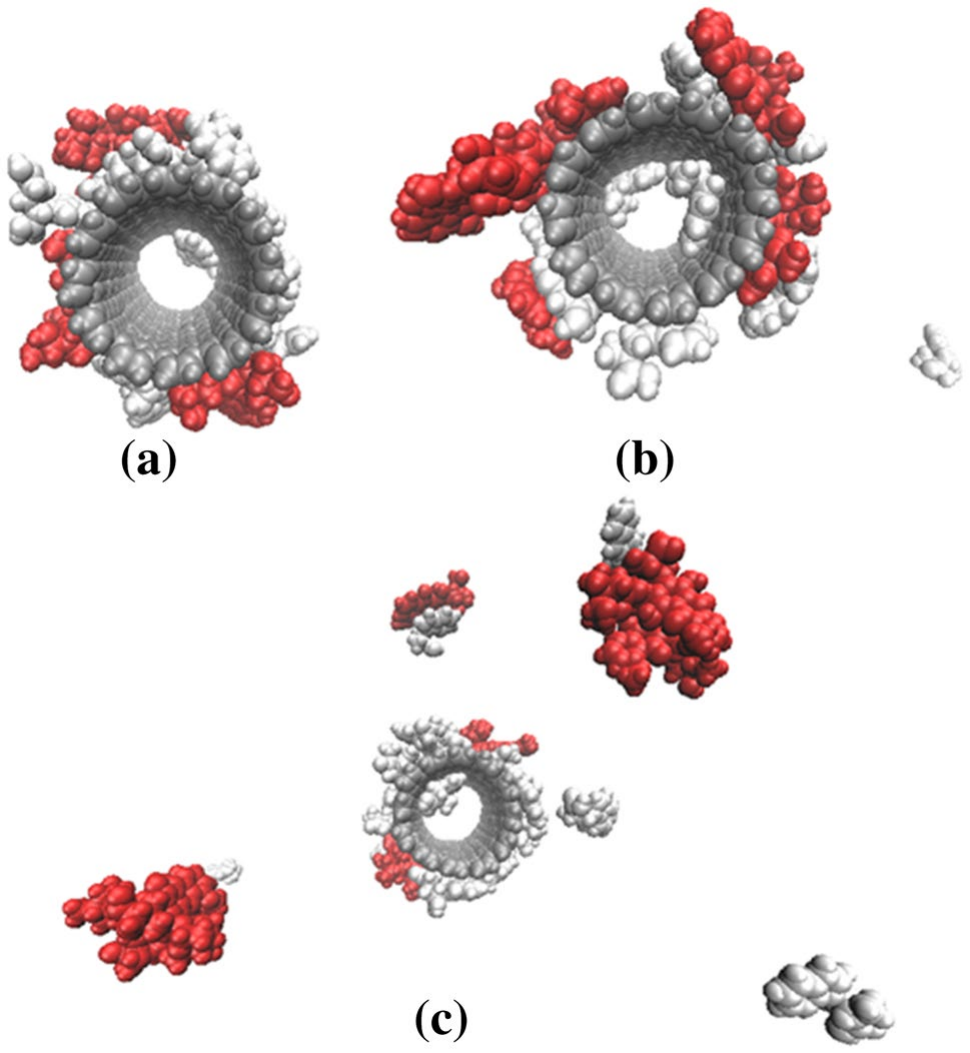
The last snapshots of drug release (red molecules) obtained by MD simulations for three studied systems. (**a**) 0 protonated state; (**b**) 10 protonated state; (**c**) 20 protonated state.

To investigate the effect of protonated Trp on the motion of DOX molecules in the studied systems, the distances between the center of mass (COM) of each DOX molecule and CNT were evaluated. The results are presented in Table 6. It can be seen that in the deprotonated state of Trp (at neutral pH), all eight drug molecules are attached to the surface of the FCNT and are in the approximate range of 0.8 to 3 nm from COM of CNT. Note that DOX molecules are adsorbed on both the CNT wall and the functional groups. Therefore, the repulsive interaction causes distance between the protonated DOX and the protonated Trp. Also, as seen in Table 6, there are some fluctuations in the calculated distance between DOX and CNT’s COM. These fluctuations for the system containing 10 DOX molecules are higher than the others. However, after 10 ns these fluctuations gradually diminished and then completely disappeared. This behavior is due to some fluctuations in the distance between DOX molecules and FCNT’s COM increasing due to repulsive interaction between protonated functional groups in FCNT and DOX, which causes deviation from the system’s minimum energy at the equilibration state. Figure 7 shows the simulation results of drug release in three stages.

**Table 6:** Variation of distance (nm) between the center of mass (COM) of CNTs and DOX molecules.

Protonated state	DOX-1	DOX-2	DOX-3	DOX-4	DOX-5	DOX-6	DOX-7	DOX-8
0	1.158	1.642	1.199	1.403	0.857	0.962	0.823	1.119
10	4.295	3.077	1.703	4.172	1.903	1.878	1.605	1.969
20	5.094	3.537	1.503	5.024	4.902	5.212	1.477	4.696

So that the system gains the equilibration state, the DOX molecules release from the FCNT surface or adjust themselves to some new adsorption locations on the FCNT surface. This phenomenon is observed at a high protonated state in which 20 Trp molecules have been protonated. At this state, 3 DOX molecules were still near to the surface, and 5 molecules also released from the surface, in the approximate range of 4 to 5 nm. From the analysis of results, it can be seen that the number of protonated states has a significant effect on drug movement. Also, according to Supplementary Tables S1, S2, and S3, with increases protonated Trp in the system, the vdW and electrostatic energies between the drug molecules and the CNT/FCNTs decrease, but energies between the drug and water molecules and between the CNT/FCNTs and water increases.

## Conclusion

In this study, we investigated the interactions of doxorubicin (DOX) as an anticancer drug with CNT/FCNTs. Tryptophan (Trp) and folic acid (FA) were used as functional groups and were attached to the CNT’s surface. The MD simulation revealed that the functionalization of single-wall CNT (SWCNT) increases the adsorption capacity of DOX molecules due to the van der Waals interaction between the FCNT and DOX molecules. Moreover, radial distribution functions (RDF) were evaluated and analyzed to understand the interactions between DOX and CNT/FCNTs. The results indicated that DOX molecules are attached to the CNT’s surface due to the formation of π–π interactions between the aromatic rings of DOX molecules and the aromatic rings of the functional groups. The obtained results manifested that the location of the DOX and Trp molecules is strongly dependent on the pH values. It was observed that the acidic pH of an aqueous solution acted as the medium, and at this pH, the number of released molecules increases by increasing protonated Trp units. One of the possible applications of this structure is its use as a drug carrier in targeted drug delivery, which needs more research and studies.

## Supplementary Information

Supplementary Information.
