# Supplementary material for: Molecular dynamics simulation study of doxorubicin adsorption on functionalized carbon nanotubes with folic acid and tryptophan
Source: Sci Rep. 2021 Dec 20;11:24210. doi: 10.1038/s41598-021-03619-8 (PMC8688492; doi:10.1038/s41598-021-03619-8)
Supplement: Supplementary file 1 — Supplementary Information. [file 41598_2021_3619_MOESM1_ESM.pdf]

Molecular Dynamics Simulation Study of Doxorubicin Adsorption on Functionalized Carbon Nanotubes with Folic acid and Tryptophan

Tahereh Arabian[a], Sepideh Amjad-Iranagh[b,*], Rouein Halladj[a,*]

[a] Department of Chemical Engineering, Amirkabir University of Technology, Tehran, Iran

[b] Department of Materials and metallurgical Engineering, Amirkabir University of technology, Tehran, Iran

*corresponding authors: Amjad_i_s@aut.ac.ir, halladj@aut.ac.ir

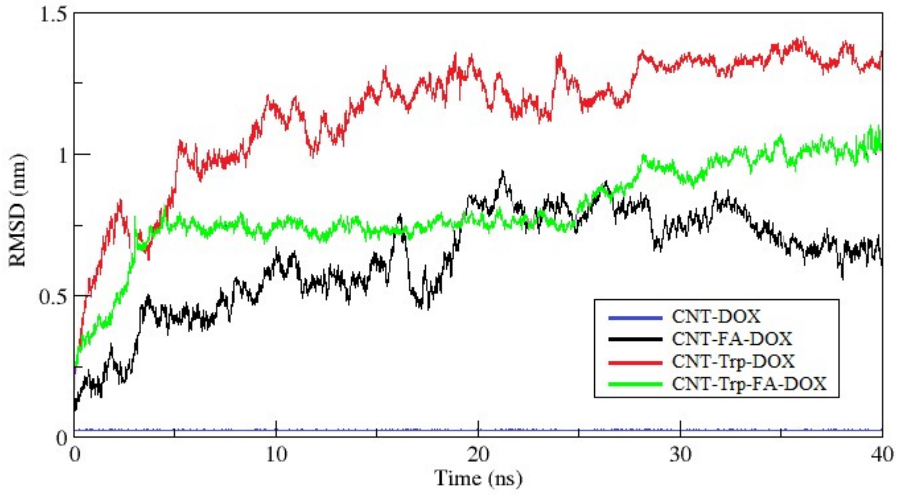

Figure S1: The root-mean-square deviation of functional groups-CNTs versus time

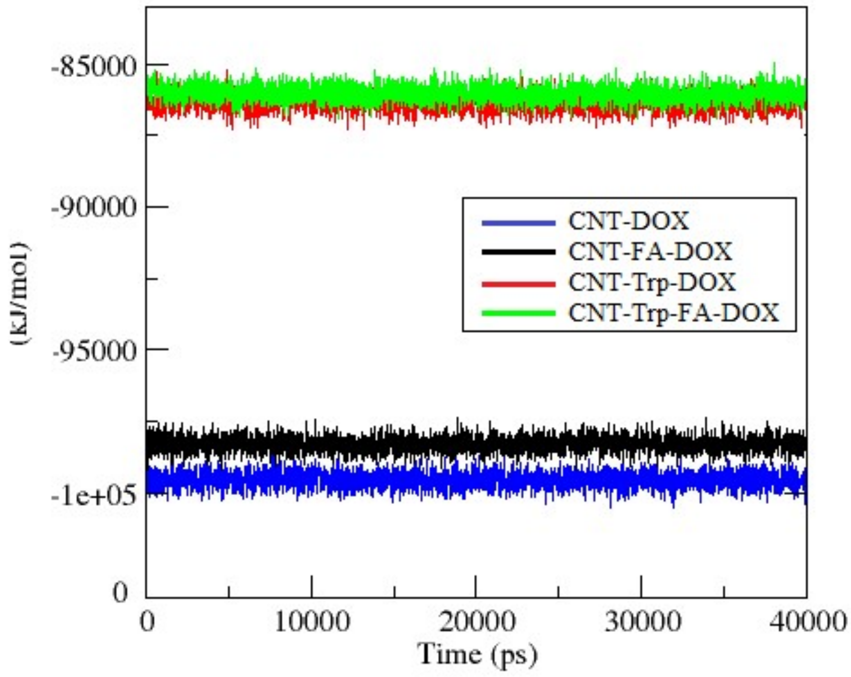

Figure S2: total energy variation versus time

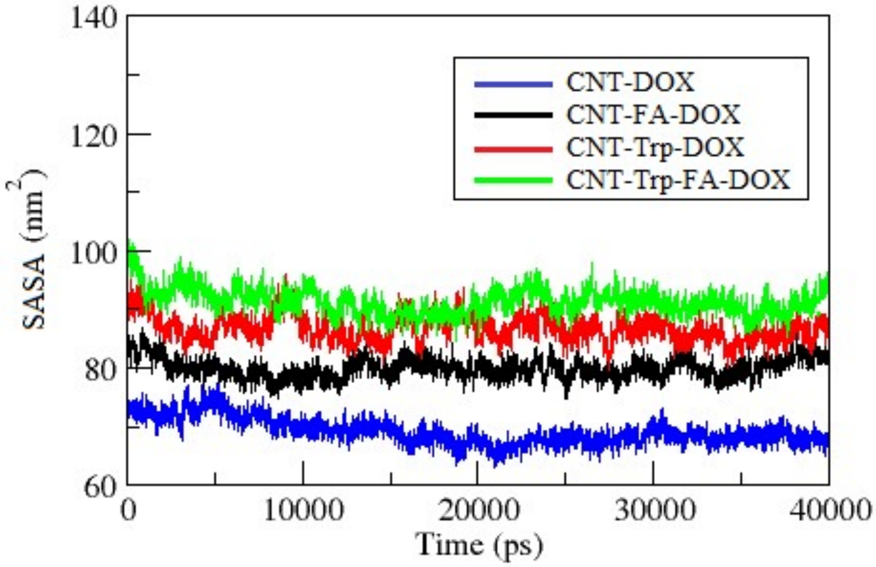

Figure S3: Solvent Accessible Surface Area variation versus time

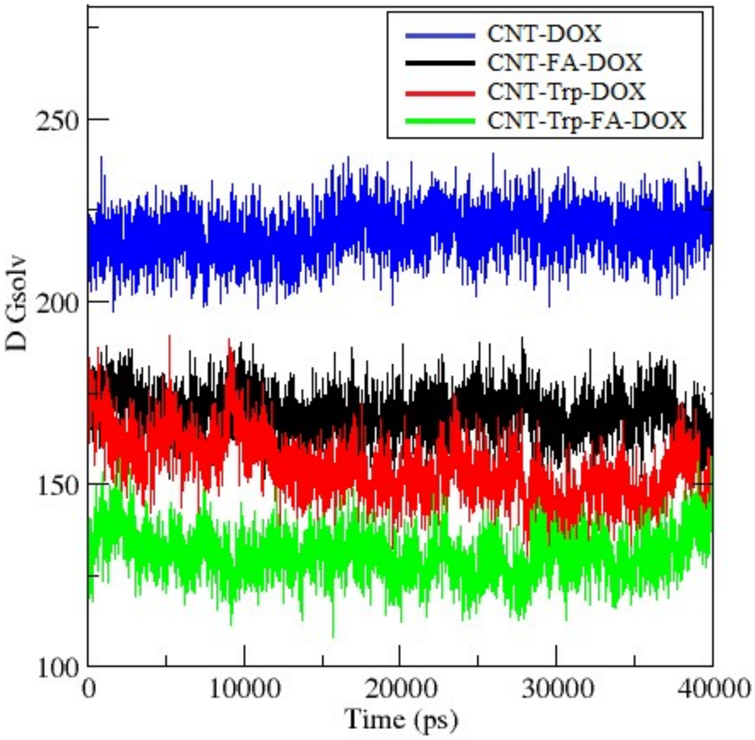

Figure S4: Solvation free energy variation versus time

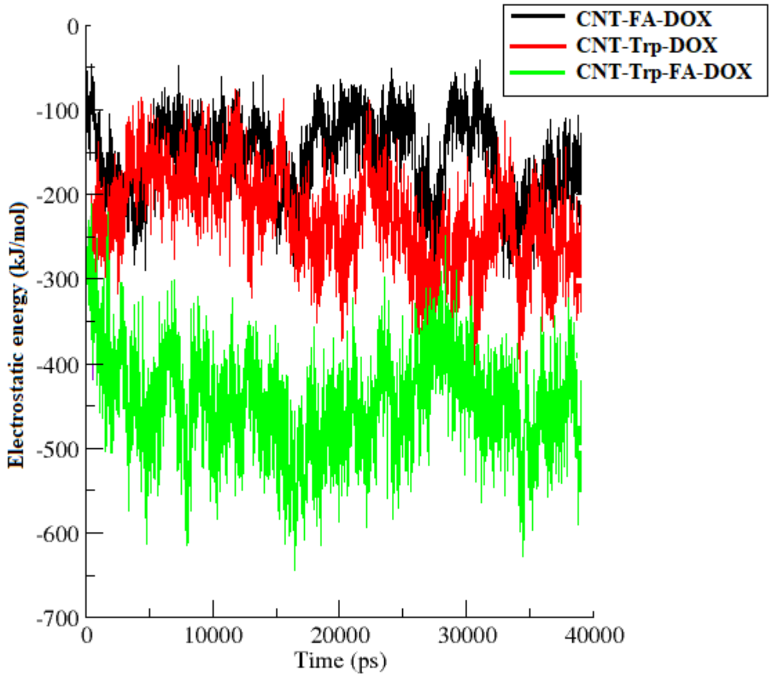

Figure S5: Electrostatic interactions between DOX molecules cariers

Table S1: Average vdW and electrostatic and total energies (kJ/mol)

protonated state	vdW (kJ/mol)	Electrostatic energy (kJ/mol)	Total energy (kJ/mol)
10	-800.26	-239.354	-1039.614
20	-391.911	-157.518	-549.429

Table S2: Average vdW and electrostatic and total energies (kJ/mol) between the DOX and water

protonated state	vdW (kJ/mol)	Electrostatic energy (kJ/mol)	Total energy (kJ/mol)
10	-905.415	-3102.66	-4008.075
20	-1003.7	-3236.81	-4240.51

Table S3: Average vdW and electrostatic energies (kJ/mol) between the carriers and water

protonated state	vdW (kJ/mol)	Electrostatic energy (kJ/mol)	Total energy (kJ/mol)
10	-2194.94	-5612.79	-7807.73
20	-2148.04	-7772.75	-9920.79
